# Preparation of Thermo-Responsive Poly(ionic liquid)s-Based Nanogels via One-Step Cross-Linking Copolymerization

**DOI:** 10.3390/molecules200917378

**Published:** 2015-09-18

**Authors:** Jing Zhang, Jingjiang Liu, Yong Zuo, Rongmin Wang, Yubing Xiong

**Affiliations:** 1 Key Laboratory of Eco-Environment-Related Polymer Materials, Ministry of Education; College of Chemistry and Chemical Engineering, Northwest Normal University, Lanzhou 730070, China; E-Mails: zhang_jing1121@163.com (J.Z.); liujingjiang214@163.com (J.L.); zuoyong_only@163.com (Y.Z.); wangrmcn@163.com (R.W.); 2 Max Planck Institute for Polymer Research, Ackermannweg 10, Mainz 55128, Germany

**Keywords:** ionic liquids, thermo-response, nanogels, cross-linking polymerization, UCST, CO_2_ conversion

## Abstract

In this study, thermo-responsive polymeric nanogels were facilely prepared via one-step cross-linking copolymerization of ethylene glycol dimethacrylate/divinylbenzene and ionic liquid (IL)-based monomers, 1,n-dialkyl-3,3′-bis-1-vinyl imidazolium bromides ([C_n_VIm]Br; *n* = 6, 8, 12) in selective solvents. The results revealed that stable and blue opalescent biimidazolium (BIm)-based nanogel solutions could be obtained without any precipitation when the copolymerizations were conducted in methanol. Most importantly, these novel nanogels were thermo-response, and could reversibly transform to precipitation in methanol with temperature changes. Turbidity analysis and dynamic light scatting (DLS) measurement illustrated that PIL-based nanogel solutions presented the phase transform with upper critical solution temperature (UCST) in the range of 5–25 °C. The nanogels were characterized using Fourier transform infrared (FTIR), thermogravimetric analyses (TGA), and scanning electron microscopy (SEM). In addition, BIm-based nanogels could also be used as highly active catalysts in the cycloaddition reaction of CO_2_ and epoxides. As a result, our attributes build a robust platform suitable for the preparation of polymeric nanomaterials, as well as CO_2_ conversion.

## 1. Introduction

In the past few decades, ionic liquids (IL) have garnered considerable attention in a variety of fields because of their excellent properties, such as vanishingly low vapor pressure, non-flammable, excellent chemical and thermal stability in the liquid state, polarity tunability, high ionic conductivity, wide electrochemical windows, *etc.* [1,2,3,4,5,6]. Meanwhile, poly(ionic liquid)s (PILs), derived from the polymerization of IL-based monomers and regarded as a distinct subclass of polyelectrolytes, can combine some unique characters of ILs with the practical properties of polymers, including processability, durability, mechanical stability. Therefore, PILs have also been collecting more and more interests in the fields of solid ion conductors, CO_2_ sorbents, dispersants, porous materials, carbon precursors, potential desalination applications, *etc.* [7,8,9,10,11].

Recently, IL-derived stimuli-responsive systems, especially thermo-responsive behavior in different solvents, have been extensively investigated and considered as one of the promising “intelligent” materials [12,13,14,15,16]. In general, thermo-responsive phase behaviors of the polymer/solvent mixtures can be divided into two general classes: that with an upper critical solution temperature (UCST) and that with a lower critical solution temperature (LCST) depending on mixing behavior of the corresponding polymer solutions upon temperature change. To date, the fabrication of IL-based thermo-responsive phase behaviors can be achieved through IL/molecule liquid mixture, polymer/IL mixture, and homo-/copolymerization of IL. Several latest reviews have summarized the recent development on the design of ILs/PILs with thermo-responsive phase behaviors in water and organic solvents [17,18,19]. As cited in these reviews, some interesting thermo-responsive IL/PIL systems were masterly explored. Both H. Ohno and J. Yuan groups have contributed great in this field [20,21,22,23]. In brief, the origin of the thermo-responsive phase behavior of ILs/PILs was inherently derived from the non-covalent interactions between IL and solvents. Given the structural diversity of the component ions, there are still many promising PIL materials that have great potential to present the thermo-responsive phase behavior. Moreover, these thermo-responsive PIL materials will provide the opportunity for discovering unprecedented phenomena and applications not previously realized for the classical neutral thermo-responsive polymers [24,25].

More recently, our group has demonstrated a facile synthesis strategy to prepare PIL-based nanogels via the conventional radical copolymerization of IL-based monomers and the cross-linkers ethylene glycol dimethacrylate (EGDMA) and divinylbenzene (DVB) in selective solvents [26,27]. Interestingly, thermo-responsive nanogels could be obtained for the first time via the copolymerization of the geminal dicationic, 1,4-butanediyl-3,3′-bis-1-vinyl imidazolium halides, and the cross-linkers described previously under the same conditions [28]. These novel PIL-based thermo-responsive nanogels can reversibly form precipitates or macrogels in methanol with the temperature changing in the range of −15 to 25 °C. Experimental studies revealed that the thermo-response of PIL-based nanogels was attributed to hydrogen bonding interactions between nanogels and methanol. Unfortunately, this strategy is infeasible when it comes to ILs which cannot form hydrogen bonding [26,27]. Thus, it is highly desired to get insight into the rules of the thermo-responsive behavior, and develop a simple approach for the preparation of thermo-responsive nanogels based PIL. To achieve this goal, we will elucidate the relationship between the thermo-response of nanogels and the structure of ionic liquid-based monomers in this paper.

## 2. Results and Discussion

### 2.1. Synthesis of Biimidazolium Salt-Based Monomers

**Scheme 1:**
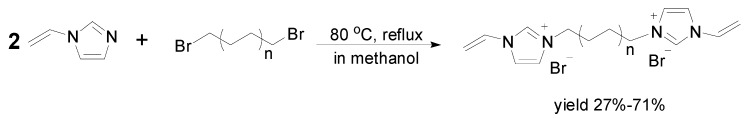
Synthetic route of biimidazolium-based ionic liquids. (*n* = 2, 3, 5).

**Figure 1:**
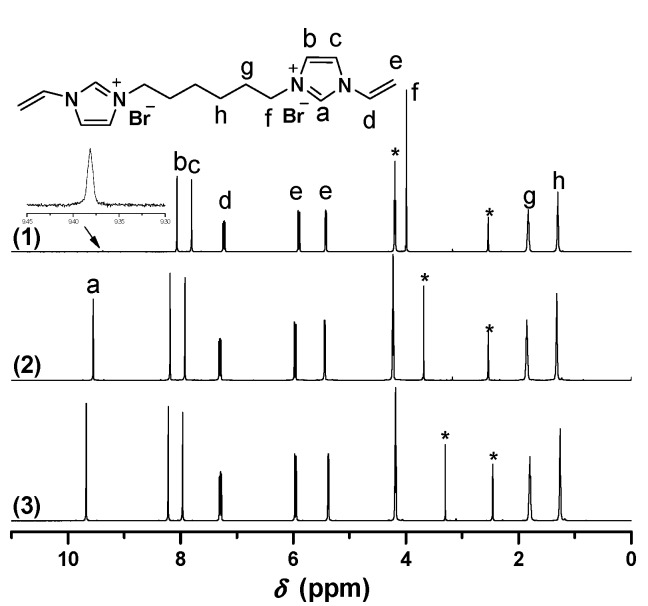
^1^H-NMR of [C_6_VIm]Br in different deuterium solvents. ((1) 0.6 mL DMSO-*d*_6_ + 0.1 mL D_2_O, 30 min later; (2) DMSO-*d*_6_ + 0.05 mL D_2_O, immediately; (3) 0.6 mL DMSO-*d*_6_). * The peak is ascribed to the solvent.

**Figure 2:**
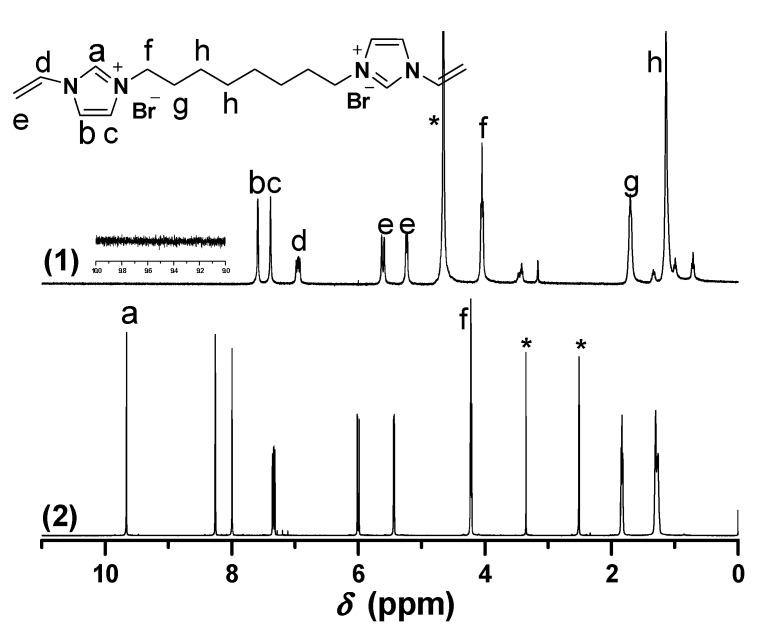
^1^H-NMR of [C_8_VIm]Br in different deuterium solvents. ((1) D_2_O; (2) DMSO-*d*_6_). * The peak is ascribed to the solvent.

**Figure 3:**
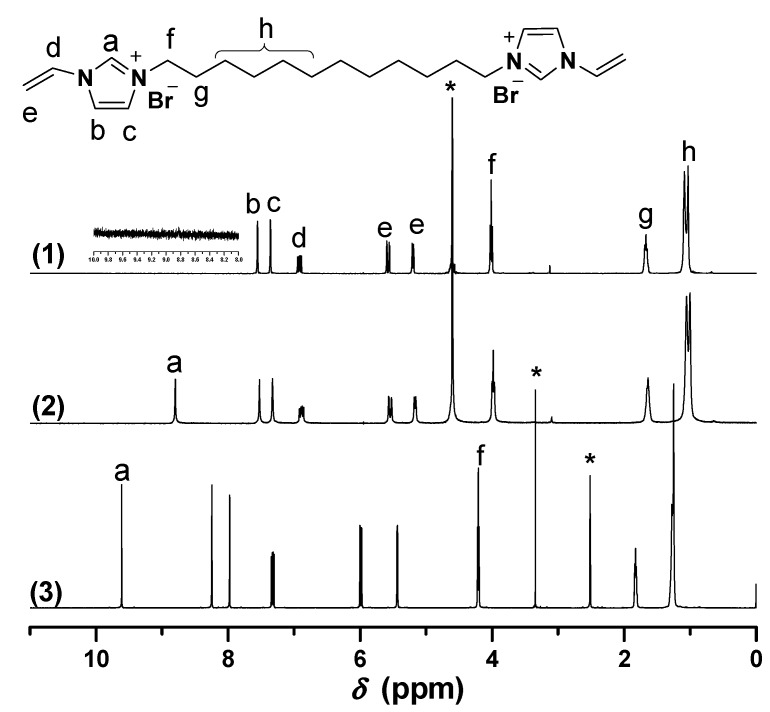
^1^H-NMR of [C_12_VIm]Br in different deuterium solvents. ((1) D_2_O, 30 min later; (2) D_2_O, immediately; (3) DMSO-*d*_6_). * The peak is ascribed to the solvent.

Biimidazolium salt (BIm)-based monomers were prepared via combination of 1-vinyl imidazole and dibromoalkane with different spacer chain lengths (as shown in Scheme 1). Their structures were characterized using ^1^H-, ^13^C-NMR, ESI-MS, and EA. The results demonstrated that all these BIm-based monomers were synthesized with acceptable yield. Likewise, ^1^H-NMR spectra of these monomers were conducted in different deuterium solvents (as shown in Figure 1, Figure 2 and Figure 3, the detail of ^1^H-NMR are shown in the supplementary materials). A prominent characteristic can be found that the signals associated with the C-2 proton in the imidazolium ring in all these BIm-based monomers can be observed clearly when the measurements were conducted in DMSO-*d*_6_. Nevertheless, these peaks cannot be observed when the measurements were conducted in deuterated water. To get more information about this feature of BIm-based monomers, deuterated water was added into DMSO-*d*_6_ in the measurement process. As illustrated in Figure 1, an obvious decrease in chemical shift can be seen after the addition of D_2_O. When more D_2_O was added, the signal associated with the C-2 proton almost disappeared. These results indicate that the C-2 proton of imidazolium ring is very active, and can exchange with the protons of polar solvents, such as water and methanol. However, the signal associated with the C-2 proton could also be observed in D_2_O when the measurement was conducted immediately after the solution was prepared (Figure 3), which indicates that the exchanging process is not very quickly. In brief, it could be confirmed that C-2 proton of the imidazolium ring in the above BIm-based monomers are active enough to form hydrogen-bond interactions with polar solvents, such as water and methanol.

### 2.2. Characterization of BIm Salt-Based Nanogels and Their Thermo-Responsive Behavior

**Scheme 2:**
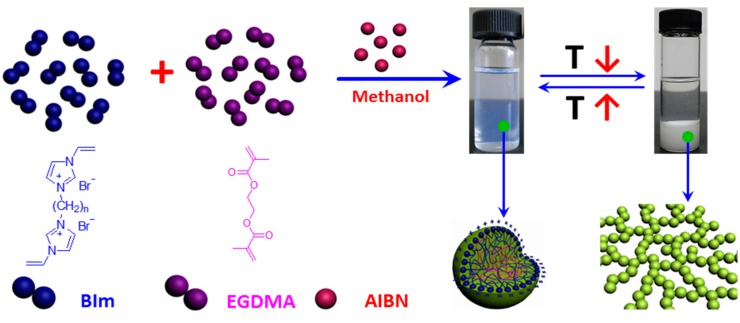
Schematic illustration of one-step synthesis of BIm-based nanogels, and their thermo-responsive behavior with temperature changes.

In our previous studies [29], it has been demonstrated that the structure of IL-based monomers play a key role in the one-step synthesis of highly cross-linked nanogel. When 1-vinyl-3-(2-methoxy-2-oxyl ethyl) imidazolium chloride was copolymerized under the same conditions, the obtained particles in the submicrometer precipitated from the solvent during the polymerization process. However, when 1,4-butanediyl-3,3′-bis-1-vinyl imidazolium bromide was copolymerized, the as-prepared nanogels could be well-dispersed in the solution and transform reversibly to precipitation or macrogel formation in methanol with the temperature changes [28]. Notably, novel thermo-responsive nanogel will promisingly be obtained by introducing hydrogen-bond interactions into IL-based nanogel. To investigate the relationship between the structure and thermo-response of nanogels in more detail, BIm-based monomers were copolymerized with the cross-linkers EGDMA and DVB via convention radical polymerization in selective solvent. As illustrated in Scheme 2, BIm-based monomers (*n* = 6, 8, and 12) can be successfully copolymerized with the cross-linkers to obtain the well-dispersed nanogels in the solution. Additionally, the nanogel solutions were very stable and could be stored without any precipitation for more than several months. The results illustrate that BIm-based monomers can stabilize the nanogels during the polymerization process. More interestingly, these BIm-based nanogels are also thermo-responsive. When the temperature decreases, the nanogels precipitate from the solvent. Inversely, the nanogels could be well-dispersed in the solvent again when the temperature increases. The performance testifies that BIm-based nanogels are of the phase behavior with UCST.

The cross-linking copolymerization with different monomers and feed ratio were conducted. Then, the size and ζ-potential of BIm-based nanogels were checked using dynamic light scattering (DLS), and the results are summarized in Table 1. It indicates that BIm-based nanogels with the size less than 160 nm can be facilely prepared via the one-step synthesis. Moreover, for the [C_6_VIm]Br + EGDMA series, hydrodynamic diameters decreased from 160 to 70 nm on decreasing the cross-linking. The particles would precipitate from the solution when the feed ratio of BIm-based monomer to EGDMA was less than 3:1. A similar trend can be observed when DVB is used as the cross-linker. This performance is reasonable because BIm-based monomers play the role of stabilizer in the cross-linking copolymerization process. Likewise [26,27,28,29], hydrodynamic diameters of these BIm-based nanogels are in a wide distribution except Entry 5. In addition, all nanogels are positively charged, and the higher feed ratio of BIm-based monomers will enhance the ξ-potential on the surface of nanogels. As a result, positive ξ-potential can improve the stability of nanogels in the solution due to electrostatic repulsion. These results strongly demonstrate that the one-step synthesis is an effective and universal method for the preparation of imidazolium-based nanogels.

**Table 1:** Size, PDI, and ζ-potential of nanogels prepared with different feed ratios in methanol.

Entry	Monomer and Cross-Linker	Feed Ratio (Molar Ratio) ^a^	D_h_ (nm)	PDI	ξ-Potential (mV)
1	[C_6_VIm]Br + EGDMA	1:1	precipitated	-	-
2	[C_6_VIm]Br + EGDMA	3:1	159	0.28	11.5
3	[C_6_VIm]Br + EGDMA	5:1	137	0.26	12.4
4	[C_6_VIm]Br + EGDMA	10:1	103	0.43	14.1
5	[C_6_VIm]Br + EGDMA	15:1	71	0.09	18.1
6	[C_8_VIm]Br + EGDMA	10:1	125	0.36	13.5
7	[C_12_VIm]Br + EGDMA	10:1	148	0.45	11.9
8	[C_6_VIm]Br + DVB	3:1	111	0.39	14.6
9	[C_6_VIm]Br + DVB	5:1	88	0.29	16.2
10	[C_6_VIm]Br + DVB	10:1	47	0.33	17.1

^a^: [C_n_VIm]Br to cross-linker; D_h_: hydrodynamic diameter; PDI: polydispersion. - No data are available.

**Figure 4:**
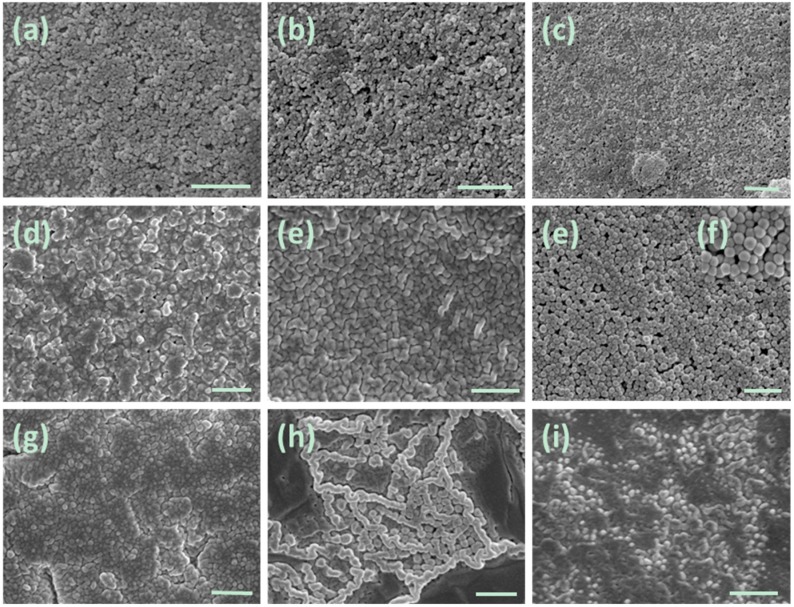
SEM images of BIm-based nanogels. ((**a**) [C_6_VIm]Br:EGDMA = 10:1; (**b**) [C_8_VIm]Br:EGDMA = 10:1; (**c**) [C_12_VIm]Br:EGDMA = 10:1; (**d**) [C_6_VIm]Br:EGDMA = 15:1; (**e**) [C_6_VIm]Br:EGDMA = 5:1; (**f**) [C_6_VIm]Br:EGDMA = 3:1; (**g**) [C_6_VIm]Br:DVB = 3:1; (**h**) [C_6_VIm]Br:DVB = 5:1; (**i**) [C_6_VIm]Br:DVB = 10:1). The scale bar is 500 nm.

The morphology of as-prepared nanogels was also examined using scanning electron microscopy (SEM). As shown in Figure 4, spherical particles with sizes of less than 200 nm can be clearly observed. Most of the particles are smaller than the corresponding hydrodynamic diameter determined by DLS (Table 1). It is because that DLS provides the data for the particles swollen in the solution, whereas SEM presents the images of dried particles. It can also be seen from Figure 4a–i that BIm-based nanogels are liable to aggregate in the dry state, which results in enlargement of some particles.

To put more light on the relationship between the structure of BIm-based monomers and the thermo-response of nanogels, phase transform behavior of BIm-based nanogels were also investigated using turbidimetry and DLS. Figure 1 shows the temperature dependence of turbidity and diameters for 5.0 wt % nanogel solutions in methanol. All these nanogel solutions are blue, opalescent, and translucent above a certain temperature, and suddenly turned cloudy below the above temperature. For example, the transmittance of nanogel solution ([C_6_VIm]Br:EGDMA = 5:1, Figure 1a) is above 80% at the temperature 18 °C. Nonetheless, the transmittance dropped promptly to below 10% when the temperature decreases to 13 °C. The discrete transition that occurred within a narrow temperature change indicates that nanogels are thermosensitive particles with an upper critical solution temperature (UCST). The thermosensitive behavior of nanogels was also confirmed by DLS measurements. The hydrodynamic diameter (D_h_) of nanogels increased rapidly from below 100 nm to micrometer level in the same temperature range. These results reveal that free nanogels were suddenly desolvated to form large aggregates, and precipitated from the solvent below UCST.

**Figure 5:**
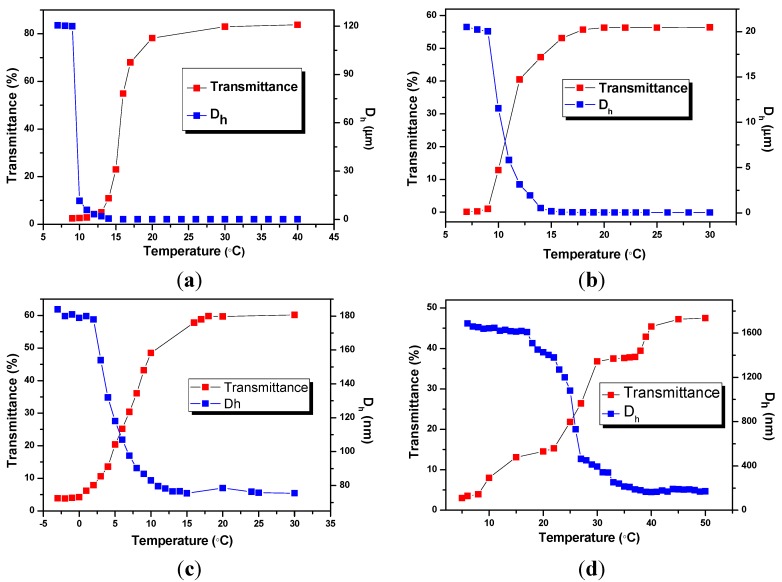
Temperature dependence of transmittance at 500 nm and hydrodynamic diameters (D_h_) for 5 wt % nanogel in methanol. ((**a**) [C_6_VIm]Br:EGDMA = 5:1; (**b**) [C_6_VIm]Br:EGDMA = 10:1; (**c**) [C_6_VIm]Br : DVB = 3:1; (**d**) [C_6_VIm]Br:DVB = 10:1).

Though BIm-based nanogels with different constitutions can present thermo-responsive behavior in methanol, marogel cannot be observed in the present system even at lower temperature (−20 °C). This performance is very different from that of nanogels with short spacer in BIm-based monomer [28]. It is probably because that H-bond interaction between imidazolium ring and methanol is not strong enough in the present system. From Figure 5c,d, It can also be inferred that more [C_6_VIm]Br in the feed will obviously enhance the UCST of the corresponding nanogel when using DVB as the cross-linker.

**Figure 6:**
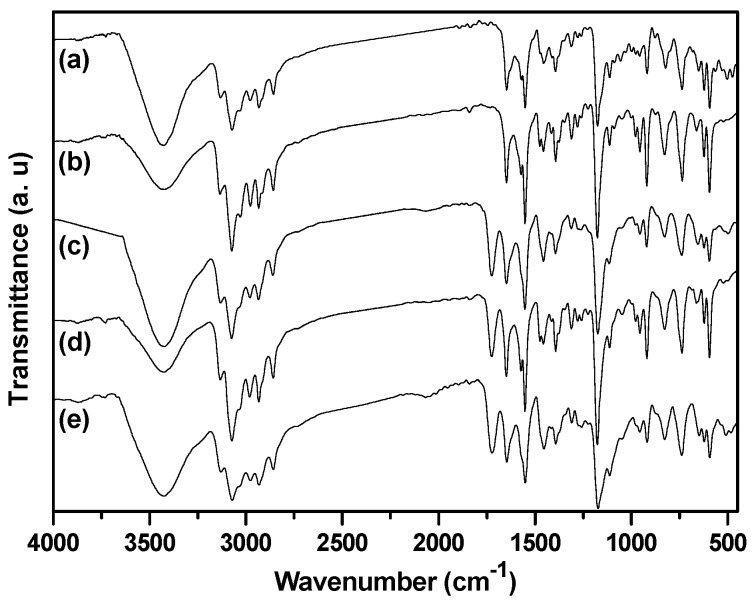
FTIR spectra of BIm-based nanogels with different monomers and feed ratio. ((**a**) [C_6_VIm]Br:DVB = 5:1; (**b**) [C_6_VIm]Br:DVB = 10:1; (**c**) [C_6_VIm]Br:EGDMA = 10:1; (**d**) [C_8_VIm]Br:EGDMA = 10:1; (**e**) [C_12_VIm]Br:EGDMA = 10:1).

**Figure 7:**
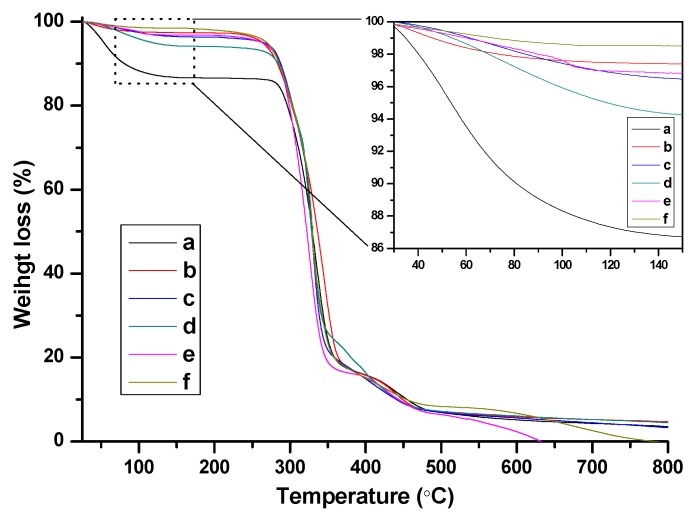
TG curves of BIm-based nanogels with different monomers and feed ratio. ((**a**) [C_6_VIm]Br:EGDMA = 15:1; (**b**) [C_8_VIm]Br:EGDMA = 10:1; (**c**) [C_6_VIm]Br:EGDMA = 5:1; (**d**) [C_6_VIm]Br:EGDMA = 10:1; (**e**) [C_12_VIm]Br:DVB = 10:1; (**f**) [C_6_VIm]Br:DVB = 3:1).

FTIR spectra of the as-prepared nanogels with different BIm-based monomers and cross-linkers are indicated in Figure 6. Some typical peaks attributed to BIm-based ILs and EGDMA/DVB copolymers can be clearly recognized, such as aromatic benzene ring (1646, 1500 cm^−^^1^, stretching vibration), aromatic imidazolium ring (1454, 1180 cm^−^^1^ stretching vibration), carbonyl group (1730 cm^−^^1^, stretching vibration), *etc*. Especially, the peak ascribed to the carbon-carbon double bond (1582 cm^−^^1^, stretching vibration) in the vinyl group of the monomers disappeared. These results demonstrate the formation of BIm-based copolymers. Thermo-stabilities of the as-prepared nanogels were measured using thermogravimetric analysis (TGA). As illustrated in Figure 7, these BIm-based copolymers are stable below 250 °C, which may be due to their highly cross-linked structure. Therefore, they can be used in some catalytic reactions at high temperatures. What is more, the weight loss before 150 °C is because of the absorbed water or solvents, and they enhanced with the increase of BIm-based monomers in the feed.

**Figure 8:**
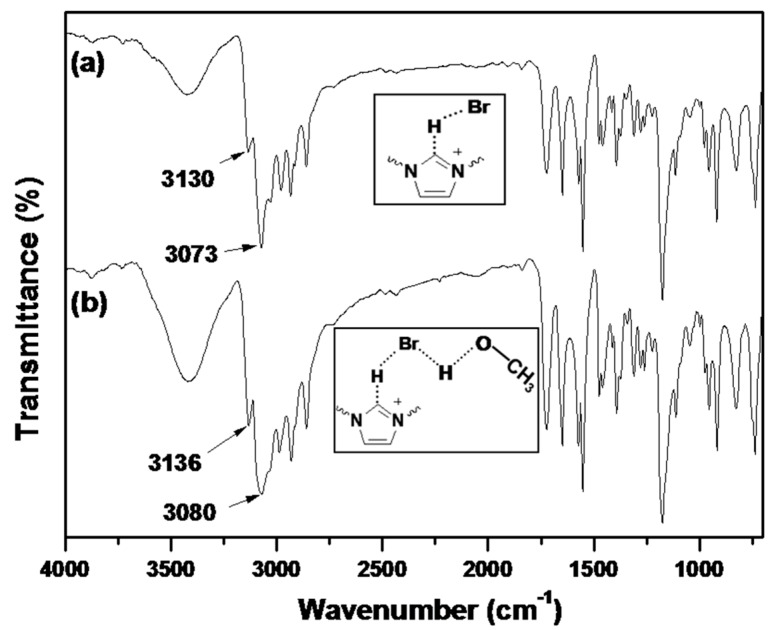
FTIR spectra of poly(EGDMA-co-[C_6_VIm]Br). ((**a**) dried sample; and (**b**) the sample containing methanol).

IR spectroscopy is an useful method to study the interactions between ILs and solvents, as well as the formation/disruption of H-bonds [30,31]. It has been reported that the IR spectra of dried imidazolium-based ILs present a prominent absorption band at 3058 cm^−^^1^, which is attributed to the C–H stretching vibration for C–H···X^−^ [32]. Upon uptake of water or other proton solvents, the peaks associated with the aromatic C–H stretching vibration and the C–H stretching vibration of the C–H···X^−^ shift to higher wavenumbers. According to Pimentel and McClellan [33], the stretching mode of an A–H moiety shifts to higher frequencies upon H-bond disruption. Infrared spectra of dried BIm-based nanogels and the ones containing little methanol are shown in Figure 8. According to the reports, the peaks at 3130 and 3063 cm^−^^1^ in the dried sample are attributed to the aromatic C–H stretching vibration and the C–H stretching vibration of C–H···Br^−^, respectively. However, the according peaks in the sample containing methanol shift to higher wavenumbers, as compared to the dried samples. This performance illustrates the disruption/diminution of the H-bond between the imidazolium ring and Br^−^, probably due to the presence of residual methanol. That is, in the presence of methanol, H-bond interactions between BIm-based nanogels and methanol may produce, and the H-bond network between nanogels and methanol results in precipitation of nanogels from the solution. However, the above H-bond interactions are weak, and can be disrupted at higher temperature. As a result, reversible phase transform is produced due to the formation/disruption of H-bond network in different temperature ranges.

### 2.3. Catalysis Performance of BIm Salt-Based Nanogels for the Cycloaddition Reaction

Catalytic performance of various BIm-based nanogels for the cycloaddition reactions of CO_2_ to epichlorohydrin (ECH) was investigated, and the results are summarized in Table 2. It can be seen that all these nanogel catalysts presented high activity (>87%) and selectivity (>89%). Higher reaction temperature, CO_2_ pressure and imidazolium salt content in the nanogels benefit to improve the yield of cyclic carbonate. Under the optimal conditions, both the activity and the selectivity of cyclic carbonate can achieve as high as 100% when catalyzed by the nanogel with the feed ratio of EGDMA to [C_6_VIm]Br of 10:1. The results above elucidated the high activity of BIm-based nanogel catalysts. It is presumably due to their nanostructure providing larger specific surface area and be dispersed easily in the substrate and product. Compared with the IL-based nanogels [26,27,34,35,36], the catalytic activity of biimidazolium-based nanogels is lower, especially the selectivity of cyclocarbonate. Only when the reaction temperature increased as high as 160 °C, the highest yield and selectivity could be achieved.

**Table 2:** Performance of BIm-based nanogel catalyst for the cycloaddition reaction of CO_2_ with ECH in different temperature ^a^.

Entry	Catalyst ^b^	Temperature (°C)	CO_2_ (MPa)	Yield (%)	Selectivity (%)
1	3:1	160	3	99.7	89.0
2	5:1	160	3	99.8	93.0
3	10:1	160	3	100	100
4	15:1	160	3	100	96.4
5	5:1	120	3	87.7	99.3
6	5:1	140	3	96.1	96.1
7	5:1	150	3	99.5	99.2
8	5:1	160	2	95.7	97.3
9	5:1	160	5	99.7	100

^a^: Reaction condition: ECH 3 mL, nanogel 0.1 g, time 6 h; ^b^: feed ratio of [C_6_VIm]Br to EGDMA.

## 3. Experimental Section

Carbon dioxide with a purity of 99.99% was provided from a commercial source. 1-Vinylimidazole (VIm, 98%), 1,6-dibromohexane, 1,8-dibromooctane, 1,12-dibromododecane, ethylene glycol dimethacrylate (EGDMA, 98%), and divinyl benzene (DVB, 98%) were brought from Aladdin Reagent Co. (Shanghai, China). The inhibitor in VIm, EGDMA, and DVB were removed through distillation on vacuum. Azobisisobutyronitrile (AIBN) was purchased from Tianjing Chemical Reagent Company (Tianjing, China), and recrystallized from methanol before use. Other reagents, such as 2,6-Ditertbutyl-4-methylphenol, methanol, diethyl ether, and acetone were A.R. grade and used without further purification.

Fourier transform infrared (FT-IR) spectra were recorded on a DIGIL FTS3000 spectrophotometer (DIGILAB, Randolph, MA, USA) using KBr tablets. ^1^H- and^13^C-NMR spectra were recorded on a Brucker AM 400 MHz spectrometer (Bruker, Faellanden, Switzerland) at 25 °C. Thermogravimetric analyses (TGA) were measured on a Perkin Elmer TG/TGA 6300 (Perkin E Itachi instruments, Norwalk, USA) at a heating rate of 10 °C min^−1^. Differential scanning calorimetry (DSC) measurements were recorded on a DSC 822e thermal analysis system (Mettler Toledo Instruments Inc., Greifensee, Switzerland) at a heating rate of ±1 °C/min with nitrogen protected (80 mL min^−1^). The morphology of nanogels was observed by scanning electron microscopy (SEM, JSM 67001F JEOL, Tokyo, Japan), and the samples were prepared by dispersing the dried nanogels in methanol under sonication at room temperature. The X-ray diffraction analysis was recorded on a Philips X’Pert using Cu Kα radiation at 40 kV (Rigaku, Tokyo, Japan). Dynamic light scattering (DLS) measurements were performed at 25 °C and a scattering angle of 90° on a commercial laser light scattering (ALV/SP-125) equipped with an ALV-5000 multi-τ digital time correlator and ADLAS DPY425 solid-state laser (output power = 22 mW at λ = 632.8 nm) (ALV-GmbH, Langen, Germany). Zeta potential values were calulated according to Smolochowski equation and each sample was analyzed in triplicate (Malvern instruments Ltd., Worcestershire, UK). The upper critical solution temperature (UCST) of thermo-sensitive nanogels was measured by turbidimetry at 500 nm using a spectrophotometer (UV-VIS Spectrometer, Lambda 20, Perkin-Elmer, Waltham, MA, USA). The elemental analyses are measured directly without any additives using a Vario EL Cube (Elementar, Hanau, Germany), and mass spectroscopy were performed using water as the solvent without additives on a micrOTOF-QII mass spectrometer (Bruker Daltonics, Billerica, MA, USA).

*1*,*6-Hexanediyl-3*,*3′-bis-1-vinyl imidazolium bromide ([C_6_VIm]Br)* 1-vinyl imidazole (5.18 g, 55 mmol) and 1,6-dibromohexane(2.79 g, 22 mmol) were dissolved in methanol (50 mL) in a round-bottom flask fitted with a condenser and N_2_ bubbler. Subsequently, the mixture was stirred at 80 °C for 48 h under a nitrogen atmosphere. After then, the reaction mixture was precipitated from diethyl ether (150 mL), and recrystallized twice from diethyl ether. The products were dried under vacuum for 24 h at R.T. [C_6_VIm]Br was obtained as white solid. Yield: 71%. [C_6_VIm]Br: ^1^H-NMR (D_2_O, 400 MHz, ppm): 7.57 (1H, s); 7.37 (1H, s); 6.93 (1H, m); 5.60 (1H, d); 5.22 (1H, d); 4.04 (2H, t); 1.70 (2H, s); 1.17 (2H, s). ^1^H-NMR (DMSO, 400 MHz, ppm): 9.66 (1H, s); 8.24 (1H, t); 7.99 (1H, t); 7.33 (1H, m); 5.99 (1H, m); 5.43 (1H, m); 4.22 (2H, t); 1.83 (2H, d); 1.31 (2H, s). ^13^C-NMR (DMSO, 100 MHz, ppm): 135.36, 128.88, 123.31, 119.23, 109.04, 49.02, 28.80, 24.77. ESI-MS (*m*/*z*): calcd. for C_16_H_24_N_4_Br_2_: 432.2, found: 432.2. Elemental analysis (%): Calculated: C, 44.46; H, 5.60; N, 12.96; Br, 36.98. Found: C, 42.94; H, 5.36; N, 12.50; Br, 39.20. M.P.: 219 °C.

*1*,*8-Octanediyl-3*,*3′-bis-1-vinyl imidazolium bromide ([C_8_VIm]Br)* 1-vinyl imidazole (0.94 g, 10 mmol) and 1,8-dibromooctane (1.36 g, 5 mmol) were dissolved in methanol (50 mL) in a round-bottom flask fitted with a condenser and N_2_ bubbler. Subsequently, the mixture was stirred at 80 °C for 48 h under a nitrogen atmosphere. After then, the reaction mixture was precipitated from diethyl ether (150 mL), and recrystallized twice from diethyl ether. The products were dried under vacuum for 24 h at R.T.Yield: 27%. [C_8_VIm]Br: ^1^H-NMR (D_2_O, 400 MHz, ppm): 8.868 (1H, s); 7.598 (1H, d); 7.398 (1H, d); 6.969 (1H, m); 5.605 (1H, m); 5.255 (1H, m); 4.057 (2H, t); 1.724 (2H, m); 1.152 (4H, s). ^13^C-NMR (D_2_O, 100 MHz, ppm): 128.39; 122.97; 119.61; 109.47; 50.07; 29.15; 28.00; 25.37. ESI-MS (*m*/*z*): calcd. for C_18_H_28_N_4_Br_2_: 460.26, Found: 460.1. Elemental analysis (%): Calculated: C, 47.15; H, 6.16; N, 12.23; Br, 34.46. Found: C, 47.40; H, 5.73; N, 11.87; Br, 35. Mp: 173–175 °C.

*1*,*12-Dodecanediyl-3*,*3′-bis-1-vinyl imidazolium bromine* *([C_12_VIm]Br)* 1-vinyl imidazole (0.94 g, 10 mmol) and 1,12-dibromododecane (1.64 g, 5 mmol) were dissolved in methanol (50 mL) in a round-bottom flask fitted with a condenser and N_2_ bubbler. Subsequently, the mixture was stirred at 80 °C for 48 h under a nitrogen atmosphere. After then, the reaction mixture was precipitated from diethyl ether (150 mL), and recrystallized twice from diethyl ether. The products were dried under vacuum for 24 h at R.T. Yield: 60%. [C_12_VIm]Br: ^1^H-NMR (D_2_O, 400 MHz, ppm): 8.80 (1H, s); 7.53 (1H, s); 7.33 (1H, s); 6.88 (1H, m); 5.55 (1H, m); 5.17 (1H, d); 3.98 (2H, t); 1.64 (2H, s); 1.03 (8H, d). ^13^C-NMR (D_2_O, 100 MHz, ppm): 134.21, 128.34, 122.94, 119.51, 109.34, 50.06, 28.68, 25.39. ESI-MS (*m*/*z*): calcd. for C_22_H_36_N_4_Br_2_: 516.36, Found: 516.2. Elemental analysis (%): Calculated: C, 51.17; H, 7.03; N, 10.85; Br, 30.95. Found: C, 50.34; H, 6.86; N, 10.62; Br, 32.18. Mp: 134.2 °C.

*Cross-linking copolymerization of [C_n_VIm]Br and EGDMA* Biimidazolium (BIm)-based nanogels were prepared by conventional radical copolymerization of [C_n_VIm]Br and cross-linkers using AIBN as initiator in selective solvents. The following example describes the typical synthesis of nanogel using 500 mol % [C_n_VIm]Br based on EGDMA. This protocol is representative of all nanogel syntheses. [C_6_VIm]Br (2.12 g, 4.9 mmol), EGDMA (0.194 g, 0.98 mmol), and AIBN (0.021 g, 0.13 mmol) were dissolved in methanol (50 mL), and the mixture was stirred at 70 °C for 48 h. After then, the mixture was precipitated from diethyl ether (150 mL), and the products were washed with THF, and diethyl ether, respectively. The products were dried under vacuum for 24 h at 50 °C. (Yield: 62.5%).

All the cycloaddition reactions were carried out in a 50 mL stainless steel reactor with magnetic stirrer and automatic temperature control system. After appropriate amounts of epoxides and catalyst were charged into, CO_2_ was introduced in, and the pressure was kept constantly till the reaction was completed. Then the reactor was heated to desired temperature. After the proper reaction time, the reactor was cooled to 0 °C by immerging into iced water. Then CO_2_ was released through a cold trap with DMF to capture the reactants and products entrained by CO_2_. The catalyst was recovered by filtration. The resulting filtrate together with the absorbent was analyzed by gas chromatography (Shimadzu GC-7A, Kyoto, Japan). The retention time of the products were compared with available authentic standards. The cyclic carbonates were characterized on a Bruker 400 MHz NMR spectrometer using CDCl_3_ as solvent at room temperature (data not shown).

## 4. Conclusions

In summary, thermo-responsive polymeric nanogels can be facilely prepared via one-step cross-linking copolymerizing of ethylene glycol dimethacrylate/divinylbenzene and biimidazolium-based monomers, 1,n-butanediyl-3,3′-bis-1-vinyl imidazolium bromides ([C_n_VIm]Br, *n* = 6, 8, 12) in selective solvents. The diameters of these nanogels are tunable via the feed ratio of BIm-monomers and the cross-linkers. It is also found that these BIm-based nanogels are thermo-responsive, and display reversible, temperature-driven phase transition in methanol. These nanogels are characterized by FTIR, TG, and SEM. Moreover, BIm-based nanogels are effective catalysts for CO_2_ cycloaddition reactions with epoxides. Such attributes make them a robust material platform suitable for a wide range of applications.

## Supplementary Materials

Supplementary materials can be accessed at: http://www.mdpi.com/1420-3049/20/09/17378/s1.
