# Supplementary material for: Preparation of Thermo-Responsive Poly(ionic liquid)s-Based Nanogels via One-Step Cross-Linking Copolymerization
Source: Molecules. 2015 Sep 18;20(9):17378–92. doi: 10.3390/molecules200917378 (PMC6332038; doi:10.3390/molecules200917378)
Supplement: Supplementary file 1 [file molecules-20-17378-s001.pdf]

Supplementary Materials

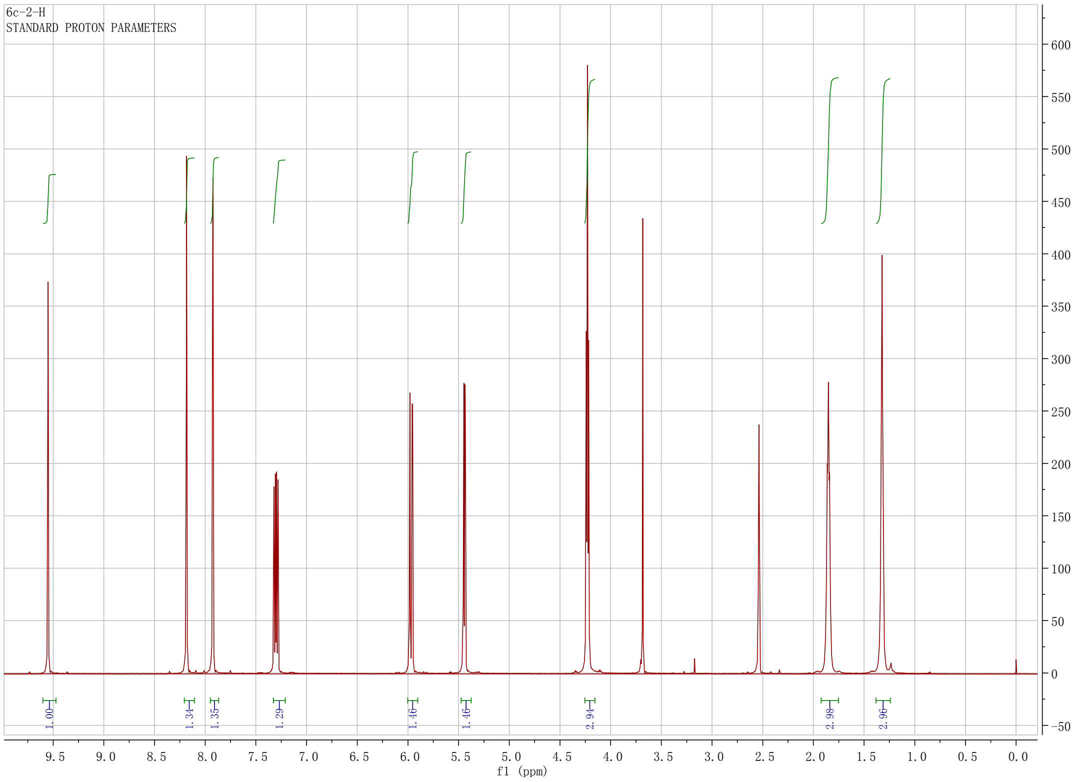

Figure S1. ^{1}H-NMR of [C_6VIm]Br in deuterium solvents. (DMSO-d_6 + 0.05 mL D_2O, immediately)

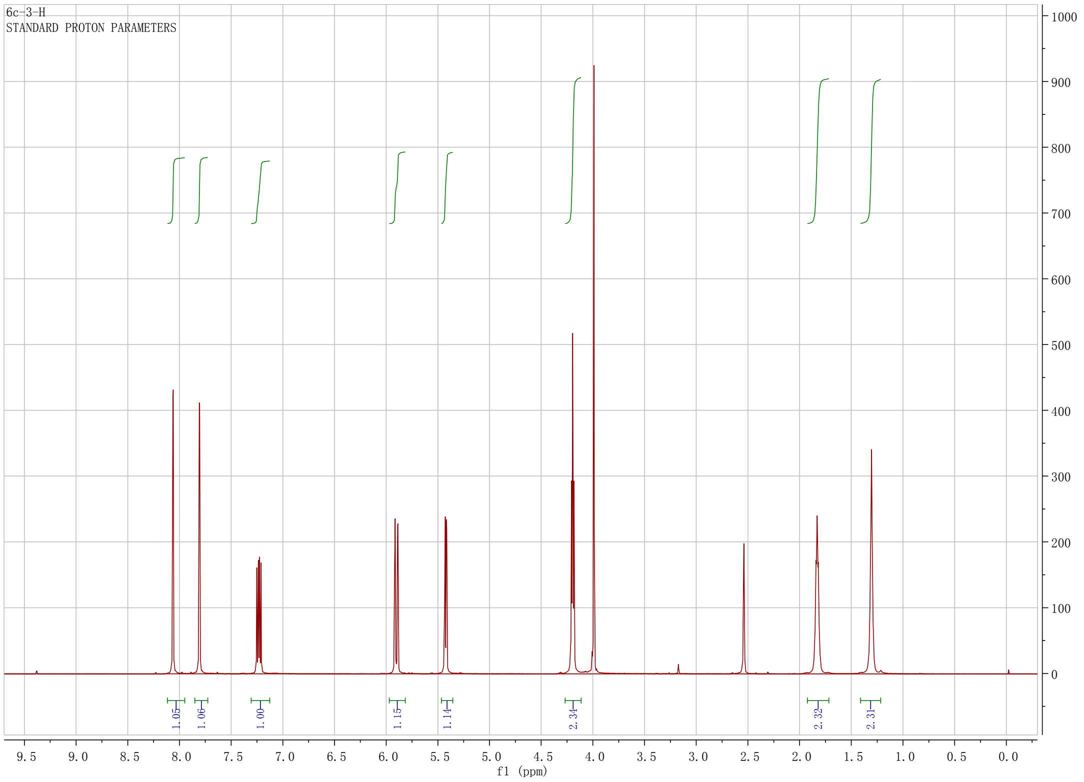

Figure S2. ^{1}H-NMR of [C_6VIm]Br in deuterium solvents. (0.6 mL DMSO-d_6 + 0.1 mL D_2O, 30 min later)

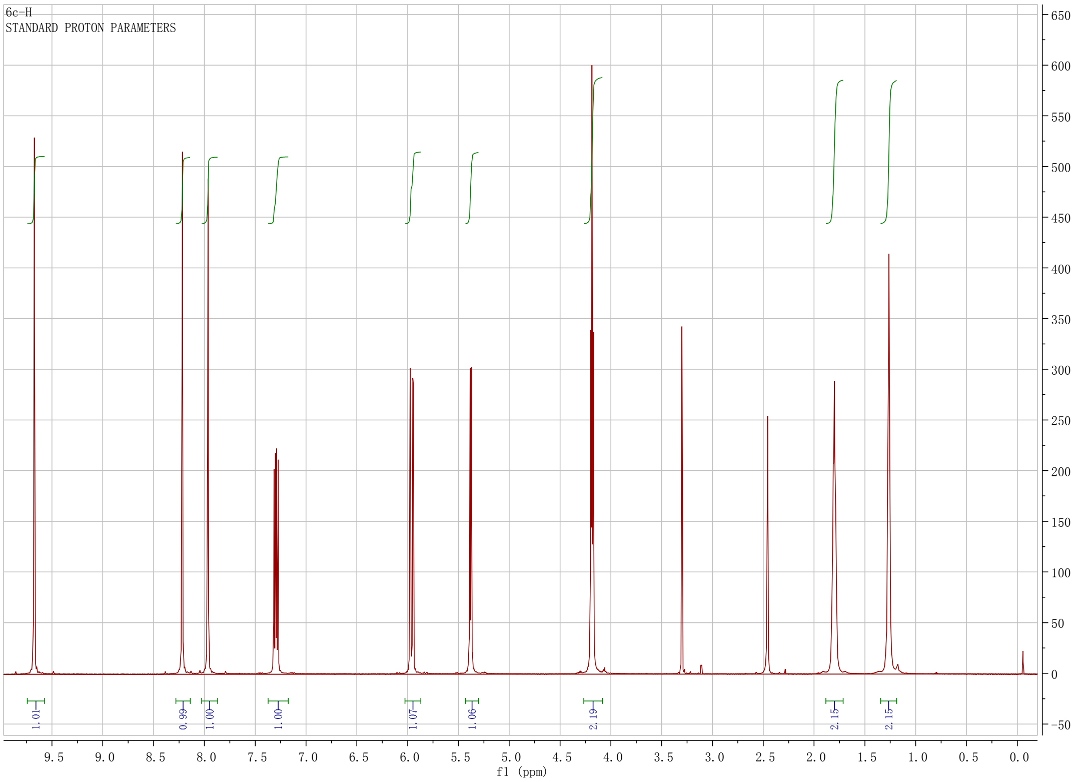

Figure S3. ^{1}H-NMR of [C_6VIm]Br in deuterium solvents. (0.6 mL DMSO-d_6).

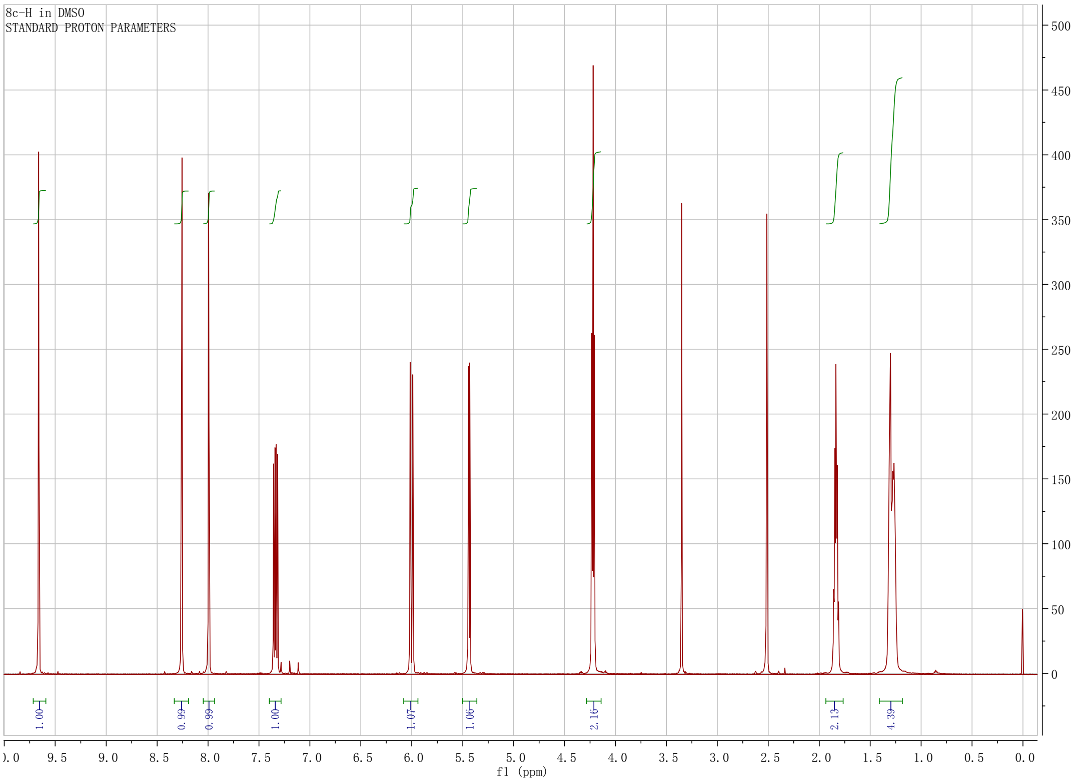

Figure S4. ^{1}H-NMR of [C_8VIm]Br in DMSO-d_6.

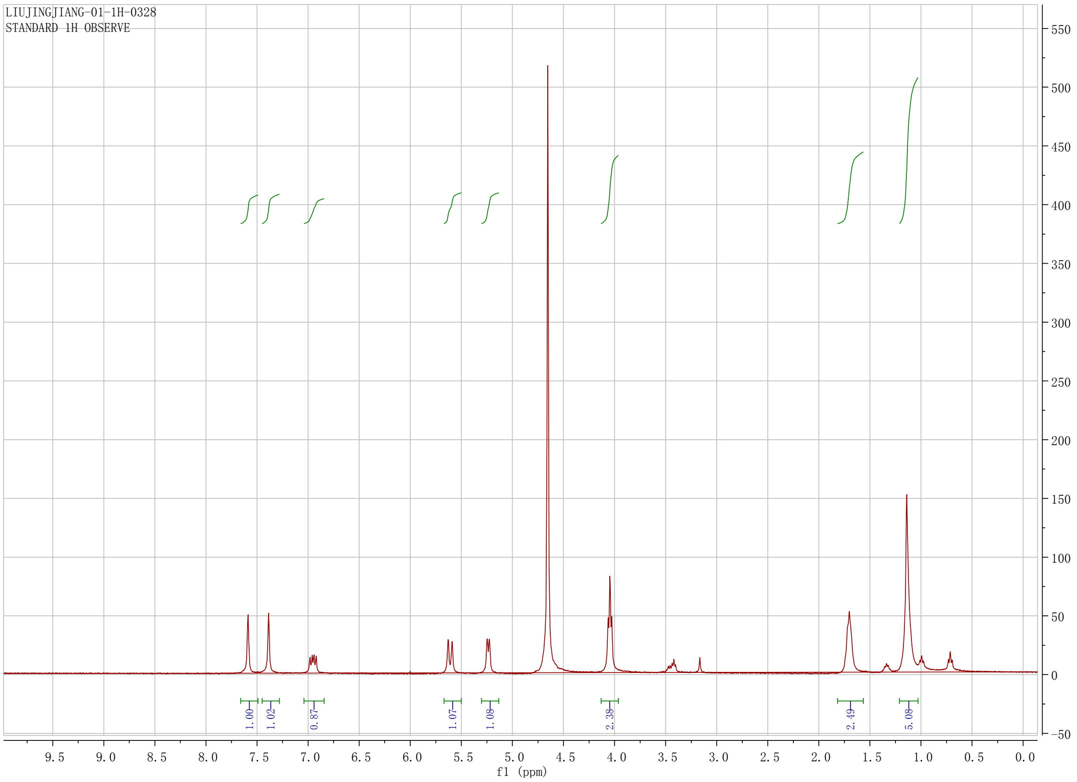

Figure S5. ^{1}H-NMR of [C_8VIm]Br in D_2O.

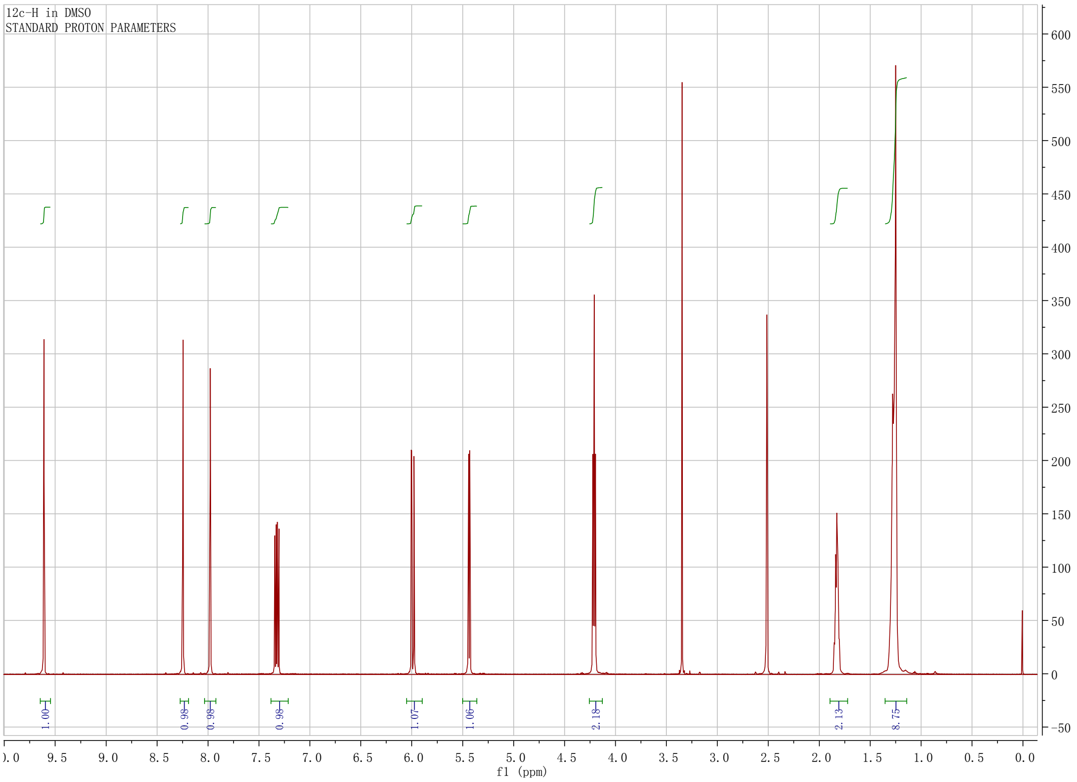

Figure S6. ^{1}H-NMR of [C_{12}VIm]Br in DMSO-d_6.

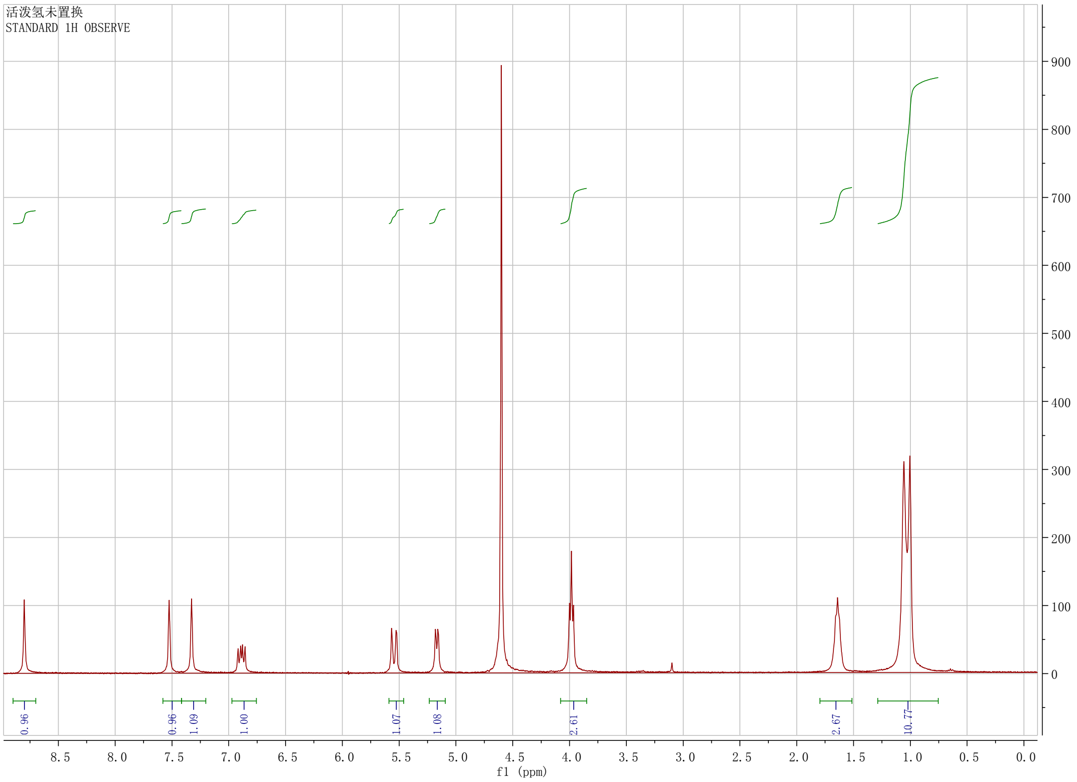

Figure S7. ^{1}H-NMR of [C_{12}VIm]Br in D_2O (immediately).

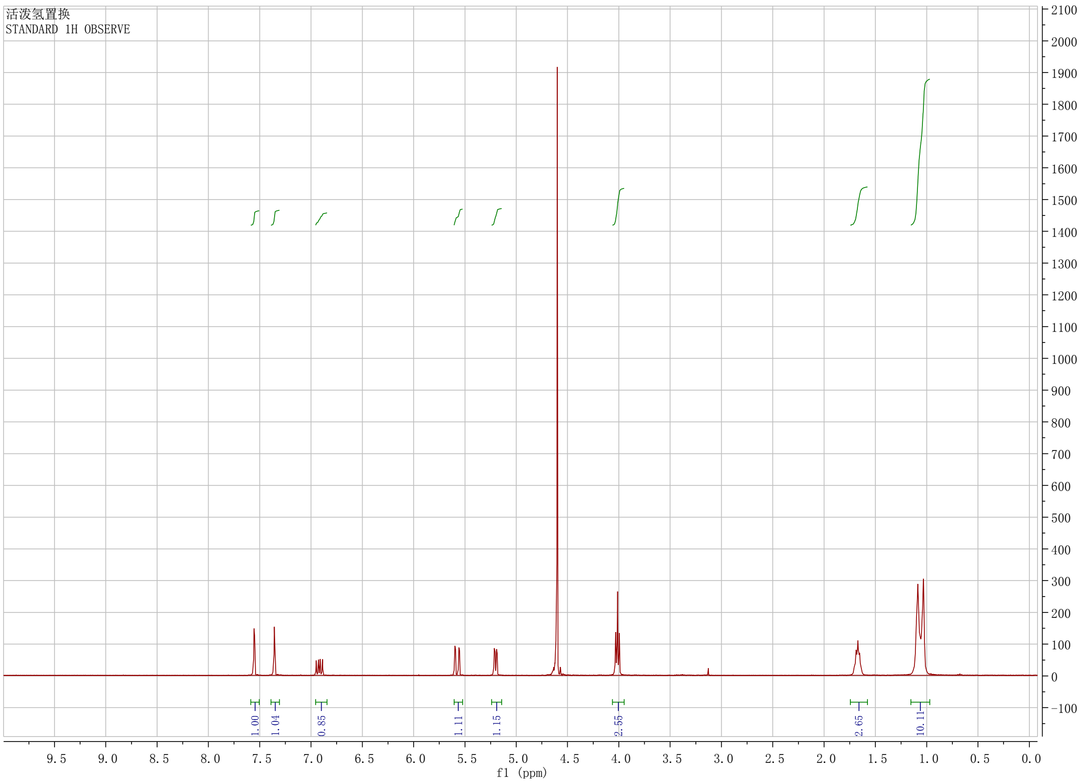

Figure S8. ^{1}H-NMR of [C_{12}VIm]Br in D_2O (30 min later).
